# The impact of hypoglycaemia awareness status on regional brain responses to acute hypoglycaemia in men with type 1 diabetes

**DOI:** 10.1007/s00125-018-4622-2

**Published:** 2018-05-12

**Authors:** Joel T. Dunn, Pratik Choudhary, Ming Ming Teh, Ian Macdonald, Katharine F. Hunt, Paul K. Marsden, Stephanie A. Amiel

**Affiliations:** 1 Division of Imaging Sciences and Biomedical Engineering, King’s College London, London, UK; 2 Diabetes Research Group, King’s College London, King’s College Hospital Campus, Weston Education Centre, 10 Cutcombe Road, London, SE5 9RJ UK; 3 Institute of Diabetes and Obesity, King’s Health Partners, London, UK; 4 Singapore General Hospital, Singapore, Republic of Singapore; 5 School of Life Sciences, University of Nottingham, Nottingham, UK

**Keywords:** Counterregulation, Hypoglycaemia, Impaired awareness of hypoglycaemia, Neuroimaging, Positron emission tomography, Type 1 diabetes

## Abstract

**Aims/hypothesis:**

Impaired awareness of hypoglycaemia (IAH) in type 1 diabetes increases the risk of severe hypoglycaemia sixfold and can be resistant to intervention. We explored the impact of IAH on central responses to hypoglycaemia to investigate the mechanisms underlying barriers to therapeutic intervention.

**Methods:**

We conducted [^15^O]water positron emission tomography studies of regional brain perfusion during euglycaemia (target 5 mmol/l), hypoglycaemia (achieved level, 2.4 mmol/l) and recovery (target 5 mmol/l) in 17 men with type 1 diabetes: eight with IAH, and nine with intact hypoglycaemia awareness (HA).

**Results:**

Hypoglycaemia with HA was associated with increased activation in brain regions including the thalamus, insula, globus pallidus (GP), anterior cingulate cortex (ACC), orbital cortex, dorsolateral frontal (DLF) cortex, angular gyrus and amygdala; deactivation occurred in the temporal and parahippocampal regions. IAH was associated with reduced catecholamine and symptom responses to hypoglycaemia vs HA (incremental AUC: autonomic scores, 26.2 ± 35.5 vs 422.7 ± 237.1; neuroglycopenic scores, 34.8 ± 88.8 vs 478.9 ± 311.1; both *p* < 0.002). There were subtle differences (*p* < 0.005, *k* ≥ 50 voxels) in brain activation at hypoglycaemia, including early differences in the right central operculum, bilateral medial orbital (MO) cortex, and left posterior DLF cortex, with additional differences in the ACC, right GP and post- and pre-central gyri in established hypoglycaemia, and lack of deactivation in temporal regions in established hypoglycaemia.

**Conclusions/interpretation:**

Differences in activation in the post- and pre-central gyri may be expected in people with reduced subjective responses to hypoglycaemia. Alterations in the activity of regions involved in the drive to eat (operculum), emotional salience (MO cortex), aversion (GP) and recall (temporal) suggest differences in the perceived importance and urgency of responses to hypoglycaemia in IAH compared with HA, which may be key to the persistence of the condition.

**Electronic supplementary material:**

The online version of this article (10.1007/s00125-018-4622-2) contains peer-reviewed but unedited supplementary material, which is available to authorised users.

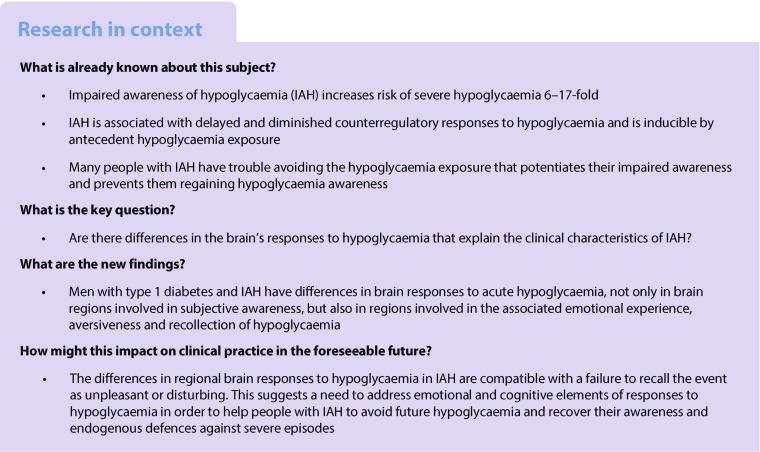



## Introduction

For people with diabetes, the main defence against severe hypoglycaemia, in which blood glucose falls too low to sustain cognitive function [1], is subjective awareness of minor episodes. Impaired awareness of hypoglycaemia (IAH) is associated with delayed and diminished counterregulatory responses to hypoglycaemia [2]. It is reported in 25–40% of people with longstanding type 1 diabetes mellitus [3, 4], and 10% with insulin-treated type 2 diabetes [5], increasing risk of severe hypoglycaemia 6- and 17-fold respectively [3–5].

The brain triggers counterregulatory neuroendocrine responses to hypoglycaemia, symptom perception, and coordination of endogenous and behavioural protective responses. Animal studies [6] have hypothesised that IAH, which is inducible by exposure to plasma glucose below 3 mmol/l [7, 8], is associated with increased brain capacity for glucose uptake. However, early studies of enhanced global brain glucose uptake in humans [9] are not compatible with the clinical picture of IAH, where deterioration of cognitive function precedes the onset of diminished and asymptomatic counterregulatory responses [10]. Early neuroimaging studies failed to confirm a global increase in glucose uptake in IAH [11].

Functional neuroimaging investigates the brain’s response to a challenge by measuring regional changes in glucose uptake and metabolism or perfusion as markers of neuronal activation. Activation of the hypothalamus, important in glucose sensing, has been described with modest decrements of blood glucose (to 4.3 mmol/l) in healthy volunteers [12]. More extensive activation, including the dorsal-medial thalamus, globus pallidus (GP) and anterior cingulate cortex (ACC), has been described in healthy people at 3 mmol/l and considered to be associated with autonomic stress responses [13]. Using [^15^O]water positron emission tomography (PET) scans repeated during induction of, maintenance of and recovery from hypoglycaemia at 2.8 mmol/l, we described activation of the thalamus and ACC, with progressive involvement of pathways involved in feeding behaviour and reward, symptom perception and aversion [14].

Less is known about the impact of diabetes and hypoglycaemia awareness (HA) status. Hormone responses to hypoglycaemia are diminished in type 1 diabetes [15], but the importance for symptoms experienced is unclear [16]. Thalamic activation has been described as enhanced in a model of IAH [17] but reduced in IAH itself [18]. A recent study compared a single measurement of global and regional cerebral blood flow (rCBF) after 45 min at 2.8 mmol/l in people with type 1 diabetes with and without IAH using arterial spin-labelling MRI. This reported a global increase in cerebral blood flow only in IAH, and failure of an enhanced regional thalamic response [19], but there are no reports of IAH-specific differences in other regional responses.

Understanding the central pathophysiology of IAH is key to its management. Although IAH can be restored by avoiding hypoglycaemia [2, 20], this can be difficult to achieve, with 8% of adults with type 1 diabetes showing low concern about hypoglycaemia despite being at high risk [21]. Cognitions around hypoglycaemia may create barriers to its avoidance [22]. We therefore extended the use of repeated [^15^O]water PET scans to measure changes in regional brain perfusion during sequential euglycaemia, hypoglycaemia and recovery in men with type 1 diabetes with and without IAH.

## Methods

### Participants

Right-handed men with type 1 diabetes aged between 20 and 50 years, with HbA_1c_ levels less than 86 mmol/mol (<10%), were recruited into two groups, based on their awareness of hypoglycaemia as defined by history and 8-item Clarke score [23]. HA was defined as good awareness of occasional hypoglycaemia, no severe hypoglycaemia in the past year and a Clarke score ≤3. IAH was defined by history, including experience of severe hypoglycaemia, and Clarke score ≥4.

The protocol was approved by the Ethics Committee of King’s College Hospital London and the Administration of Radioactive Substances Committee (ARSCAC, Health Protection Agency, Didcot, Oxfordshire, UK). All participants gave written informed consent.

### Protocol

Scans were performed at the PET Imaging Centre, St Thomas’ Hospital, London, using a GE Discovery ST PET/computerised tomography (CT) scanner (GE Medical Systems, Milwaukee, WI, USA) with a 15.8 cm axial field of view. Participants were admitted the evening before the study. Two intravenous cannulae were inserted for infusion of soluble insulin (Actrapid; NovoNordisk, Copenhagen, Denmark) in a 4% (vol./vol.) saline (154 mmol/l NaCl) solution of autologous blood; blood was sampled hourly. Participants fasted after their evening meal, sips of water being allowed, and omitted their evening background insulin dose. To achieve normoglycaemia without hypoglycaemia, plasma glucose was maintained between 4 and 7 mmol/l overnight by adjusting the insulin infusion.

In the morning, the left radial artery was cannulated. After at least 20 min, each participant rested supine on the scanner trolley, in a headrest with a forehead positioning strap. Once in the scanner, the head position was checked using gantry lasers. A new primed-continuous infusion of soluble insulin was started, at a maintenance rate 1.5 mU kg^−1^ min^−1^. Four minutes later, an infusion of 10% (wt/vol.) glucose (Baxter Healthcare, Thetford, Norfolk, UK) was started and was adjusted using 5 min arterial plasma glucose readings (YSI 2300 Stat Plus; Yellow Springs Instruments, Yellow Springs, OH, USA). The target plasma glucose level was 5 mmol/l for 40 min, with a reduction over 20 min to 2.6 mmol/l, maintenance at 2.6 mmol/l for 45 min, and restoration to 5 mmol/l (over 15–20 min) for 30 min. The insulin was then stopped, and the participant withdrawn from the scanner. He was then given lunch, with his usual subcutaneous fast-acting insulin pre-meal dose and, if a morning dose of basal insulin had been omitted, a reduced basal insulin dose to provide cover until the evening dose. The glucose infusion was reduced and plasma glucose monitored until concentrations were spontaneously maintained. The cannulae were then removed. Participants were advised about monitoring and dosing to minimise risk of hypoglycaemia over the next 24 h.

### Scanning protocol

This was as previously reported [14]. In brief, the brain was localised in the PET view field using a planar CT scout. A low-dose CT scan was acquired to correct attenuation in subsequent PET scans. Head position was checked, and 3 min [^15^O]water PET scans were made at 10 min intervals. For each scan, 350 MBq [^15^O]water in 10 ml sterile water was manually injected intravenously over 10 s. Three scans were acquired during euglycaemia: one during the fall in glucose, five during the hypoglycaemic phase and three during recovery. Scans were acquired in 3D mode, reconstructed to a single static frame using the 3D FORE algorithm [24], and 2D-filtered back-projection with scatter correction and CT-based correction of attenuation. To minimise movement artefacts, each participant’s CT scan was realigned to each PET scan using the rigid-body registration algorithm in the Statistical Parametric Mapping 2 program (SPM2; www.fil.ion.ucl.ac.uk/spm, accessed 15 March 2013). The realigned CT was used to correct the attenuation in the PET reconstruction.

### Assessment of physiological responses

After scanning, arterial blood was taken to measure counterregulatory hormones. The participant was asked verbally to rate (from 1 to 7) 13 hypoglycaemia-associated symptoms (see below) [25].

### Biochemical analyses

Blood was kept on ice, spun, separated and flash-frozen on dry ice until storage. A volume of 1 ml was added to 30% (wt/vol.) polyethylene glycol for free insulin radioimmunoassay (Diagnostic Systems Laboratories, London, UK). Blood (3 ml) for catecholamines was taken into heparinised tubes containing 15 μl sodium metabisulphite; plasma was separated at the time of study, stored at −80°C and analysed by radioimmunoassay [14].

#### Symptom scores

Scores for sweating, shakiness, anxiety, warmth, palpitation and tingling were summed as autonomic. Scores for dizziness, irritability, difficulty in speaking, confusion, lack of energy, drowsiness and poor concentration were totalled as neuroglycopenic.

### Statistical analyses of non-imaging data

Age, BMI, HbA_1c_ and duration of diabetes were compared between groups using independent sample *t* tests, and Clarke scores by Mann–Whitney *U* testing. Hormonal and symptom responses to hypoglycaemia and impact of awareness status were examined by calculating the main and interaction effects of scan and group on each variable using a repeated measures linear mixed model (first-order autoregressive covariance structure). To assess the effect of hypoglycaemia, baseline values for each variable were calculated by averaging data from scans 1–3. A summary statistic of response to the total hypoglycaemic period was calculated as the incremental AUC (iAUC) of the response in scans 4–9. The effect of hypoglycaemia was tested using a one-sample *t* test of the iAUC if the mixed model revealed significant scan or interaction effects. To assess differences between the two groups, the baseline and iAUC measures were used in two-sample *t* tests if the mixed model revealed significant group or interaction effects. Analyses were performed using SPSS software version 24 (www-01.ibm.com/software/uk/analytics/spss/).

### Neuroimaging analysis

The analytic program was chosen to allow interrogation of the data without preconceptions of the brain regions that might respond to hypoglycaemia in people with type 1 diabetes or respond differently between HA and IAH. SPM2 was used to preprocess and analyse the PET data. Image processing and analysis of regional perfusion were performed automatically and identically in each participant or group, removing the need for blinding. PET images were transformed into standard anatomical space conforming to the standard Montreal Neurological Institute (MNI) space using the [^15^O]water PET template supplied with SPM2, masked to exclude the scalp and smoothed using a Gaussian kernel (full width at half maximum [FWHM] = 6 mm).

Within SPM2, each image was spatially normalised to its whole-brain mean using SPM2, which performs an initial affine registration followed by a basis function method non-linear registration [26] and calculates regional perfusion relative to the whole-brain mean. SPM2 calculates the mean image intensity across the whole brain, without segmentation, using its default threshold method (mean of the overall mean × 0.8), and uses integrated activity over time (here 3 min), which is proportional to rCBF [27].

For analysis, scans were grouped as: euglycaemia/baseline (scans 1–3), early hypoglycaemia (scans 4–6), established hypoglycaemia (scans 7–9) and recovery (scans 10–12). Two-way repeated measures ANOVA was used to investigate the main effects of stage of hypoglycaemia (early, late or recovery vs baseline), group and interactions [28].

Regions of significant effects were calculated using a voxel-level *p* value <0.001 and cluster sizes (*k*) ≥100 voxels; these were recalculated at *p* < 0.005, *k* ≥ 50 voxels to examine for smaller differences where more significant differences had been excluded. *t* values <2 (or <−2) indicate significance with >95% confidence. Clusters were localised using the Tziortzi atlas [29].

## Results

### Participants

Of the 17 men with type 1 diabetes recruited, nine had intact HA. These nine individuals were aged 37.6 ± 9.3 years and had a diabetes duration of 14.3 ± 12.3 years, BMI, 23.4 ± 3.4 kg/m^2^ and HbA_1c_, 58.5 ± 12.4 mmol/mol (7.5 ± 1.3%) (all means ± SD). They reported good awareness of hypoglycaemia, with a median Clarke score of 2 (range 1–3) and no severe hypoglycaemia in the past year. The other eight men (means ± SD: age, 36.4 ± 7.8 years; diabetes duration, 27.1 ± 11.9 years; BMI, 26.6 ± 1.4 kg/m^2^; HbA_1c_, 57.7 ± 9.3 mmol/mol [7.3 ± 0.8%]) had IAH, with a median Clarke score of 5.5 (range 4–6); all had a history of severe hypoglycaemia. The IAH and HA groups did not differ in terms of age (*p =* 0.79), duration of diabetes (*p =* 0.07) or HbA_1c_ (*p =* 0.89) but had slightly a greater BMI (*p =* 0.02). By design, Clarke scores were significantly different between groups (*p* < 0.001).

### Plasma insulin, glucose, symptoms and counterregulatory hormones

**Fig. 1:**
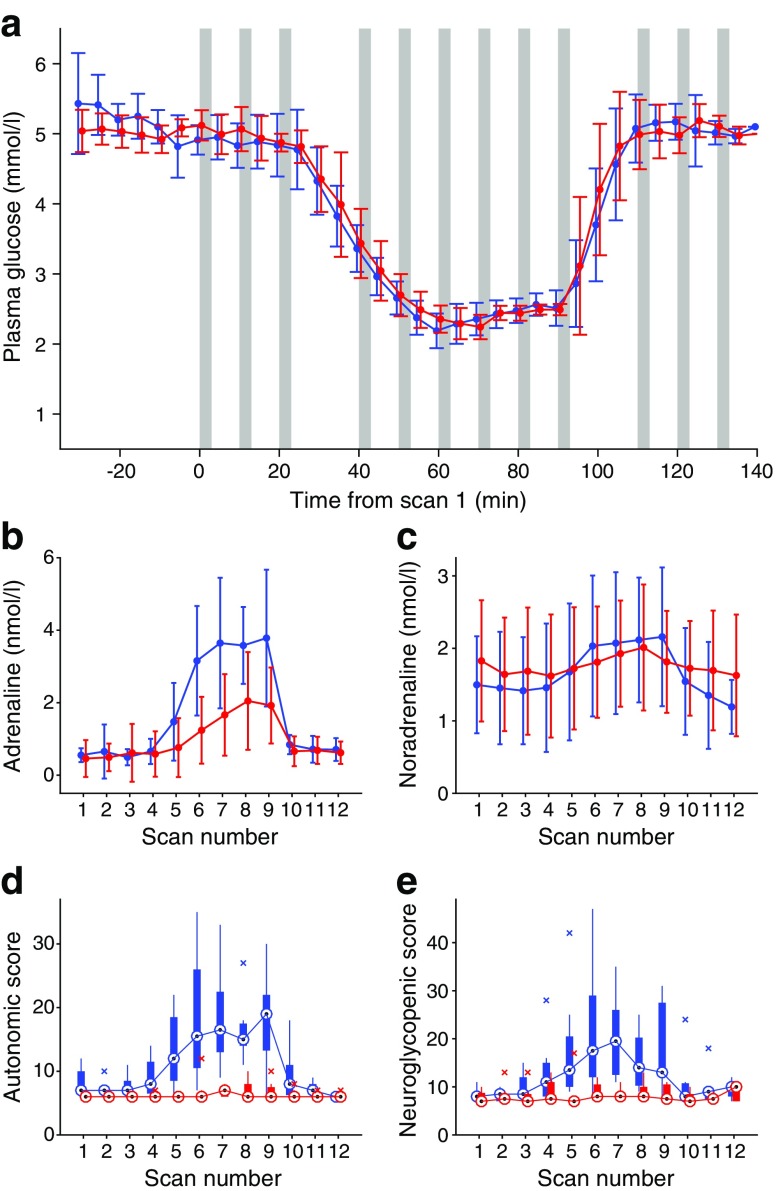
Plasma glucose, catecholamine and symptom responses. Blue lines, HA; red lines, IAH. (**a**) Plasma glucose levels (mean ± SD). Grey bars represent scan time points. iAUC was not significantly different between groups. (**b**, **c**) Mean ± SD at each scan for (**b**) plasma adrenaline (iAUC, *p =* 0.007 between groups) and (**c**) plasma noradrenaline (significant response to hypoglycaemia in HA only (*p* < 0.009); no significant difference in iAUC between groups. (**d**, **e**) Box plots showing median (circles), upper and lower quartiles (box), range (vertical lines) and outliers (crosses) for (**d**) autonomic symptom scores and (**e**) neuroglycopenic symptom scores. For both (**d**) and (**e**), difference between groups, *p =* 0.002

During the studies, steady-state free plasma insulin was (mean ± SD) 476.4 ± 138.48 and 487.9 ± 225.3 pmol/l for the HA and IAH group, respectively (*p =* 0.89). The hypoglycaemia achieved (Fig. 1a) did not differ between groups (2.4 ± 0.1 and 2.4 ± 0.1 mmol/l, respectively; *p =* 0.7); there were no significant differences in starting glucose level, or glucose concentration during recovery. Mean glucose levels were: scan 4, 3.4 ± 0.2 vs 3.5 ± 0.4; scan 5, 2.7 ± 0.2 vs 2.7 ± 0.3; and scan 6 2.2 ± 0.3 vs 2.3 ± 0.2 (all *p* > 0.05). Hormone concentrations did not differ between the groups during euglycaemia. Adrenaline (epinephrine) and noradrenaline (norepinephrine) (Fig. 1b, c) showed significant main effects of scan number (see also electronic supplementary material [ESM] Table 1), with a significant interaction effect of scan and group for adrenaline levels. Post hoc tests of effect of hypoglycaemia using iAUC revealed significant responses in HA for adrenaline (*p* < 0.001) and noradrenaline (*p =* 0.009). The adrenaline response with IAH was significant (*p =* 0.003) but significantly lower than for HA (*p* for comparison *=* 0.007). There was a significant noradrenaline response only in the HA group (*p =* 0.009). The iAUC for cortisol did not reach significance in either group, with a trend towards significance for HA (*p =* 0.075; see ESM Table 1). The iAUC for growth hormone was significant in both groups (HA, *p =* 0.035; IAH, *p =* 0.006; see ESM Table 1).

### Symptom responses

Significant main effects for scan, group and interaction were revealed in autonomic and neuroglycopenic total scores (Fig. 1d, e; ESM Table 1). The HA group showed significant responses (iAUC) for both scores (422.7 ± 237.1, *p =* 0.001 and 478.9 ± 311.1, *p =* 0.002, respectively). The IAH group showed a small response for autonomic scores (26.2 ± 35.5; *p =* 0.038), significantly lower than the response with HA (*p =* 0.002), and no significant neuroglycopenic symptom response (34.8 ± 88.8, *p =* 0.15 from baseline, *p =* 0.002 vs HA group). Baseline autonomic scores were slightly higher for HA than IAH (*p =* 0.024), with a mean difference in score of 1.5, which was small in comparison with the mean increase of 10.5 seen during hypoglycaemia.

### Neuroimaging data

#### Regional brain responses to acute hypoglycaemia in type 1 diabetes with preserved HA

**Fig. 2:**
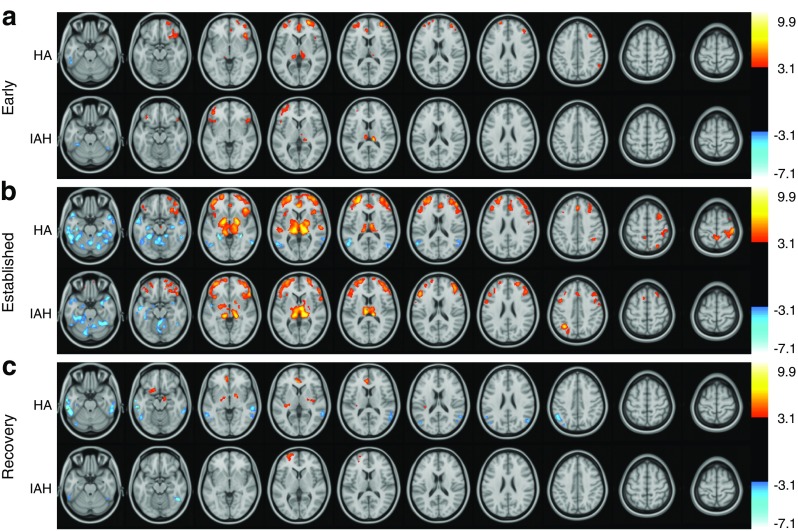
SPM2 results showing significant (voxel level *p* < 0.001, k ≥ 100 voxels) regional changes in brain perfusion compared with baseline euglycaemia during (**a**) early hypoglycaemia, (**b**) established hypoglycaemia and (**c**) recovery from hypoglycaemia measured with repeated [^15^O]water PET scans in men with IAH and without (HA). For analysis, scans were grouped as euglycaemia (scans 1–3), early hypoglycaemia (scans 4–6), established hypoglycaemia (scans 7–9) and recovery (scans 10–12). Significant increases in rCBF are shown in red-yellow, and decreases are shown in blue-white. *t* values are shown in the right-hand scale. Scans were overlaid onto an MRI scan in Montreal Neurological Institute standard space (greyscale)

The response to early hypoglycaemia across HA participants (Fig. 2a, ESM Table 2) included a regional increase in perfusion (compared with baseline) in the thalamic pulvinar bilaterally, bilateral dorsolateral frontal (DLF) cortices, right insular cortex and ACC; a decrease in perfusion was seen in the left inferior temporal gyrus. As hypoglycaemia progressed (Fig. 2b, ESM Table 3), the activation area became more extensive, with additional activation (vs baseline) in the following: posterior, middle and anterior thalamus and GP; bilateral insula and frontal opercula; frontal cortex including the DLF cortex bilaterally and lateral orbital cortex; ACC; and precuneus and right angular gyrus/supramarginal gyrus/superior temporal gyrus. Perfusion was reduced in established hypoglycaemia bilaterally in the parahippocampal and posterior parietal cortex, inferior temporal gyri and parts of the cerebellum including the vermis. During recovery (Fig. 2c, ESM Table 4), regions including the ACC, GP, right insula and left precuneus showed activation; there was persisting deactivation in the inferior temporal and posterior parietal gyri, and new activation of the amygdala.

#### Impact of awareness of hypoglycaemia on regional brain responses to hypoglycaemia

**Fig. 3:**
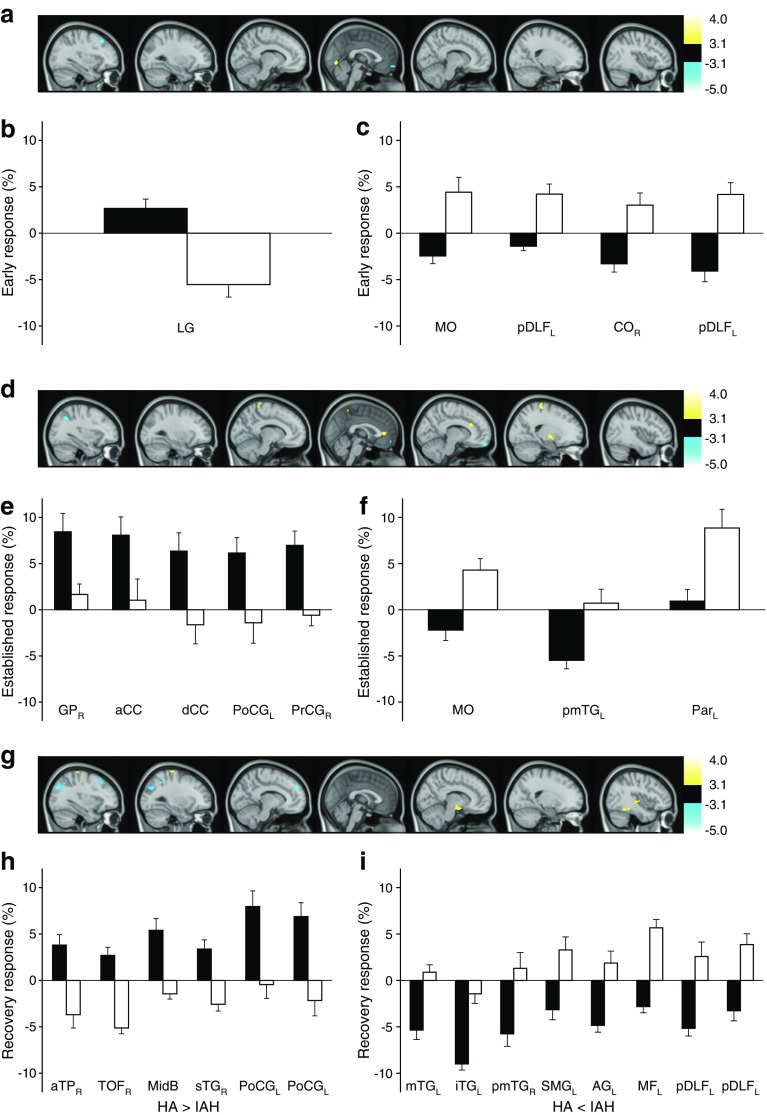
SPM2 results for comparisons of HA and IAH responses to hypoglycaemia (see Table 1). (**a**, **d**, **g**) Images showing regions with significantly different responses between HA and IAH to early (**a**), established (**d**) and recovered (**g**) hypoglycaemia (change from baseline). Changes were detected using SPM2 statistical thresholds set at voxelwise *p* < 0.005 and *k* ≥ 50 voxels. No clusters were seen with stricter thresholds (*k* ≥ 100 voxels, *p* < 0.001). Yellow clusters show regions where response is significantly more positive for HA than IAH; blue clusters show regions where response is more negative in HA than IAH. *t* values are shown in the right-hand scale. Associated bar charts show the mean and SE of group responses for whole clusters; all between-group differences were significant, as defined above. (**b**, **e**, **h**) Data for clusters where response is more positive for HA than IAH in early (**b**), established (**e**) and recovered (**h**) hypoglycaemia. (**c**, **f**, **i**) Data for clusters where response is more negative for HA than IAH in early (**c**), established (**f**) and recovered (**i**) hypoglycaemia. Black bars, HA; white bars, IAH. a, anterior; AG, angular gyrus; CC, cingulate cortex; CO, central operculum; d, dorsal; i, inferior; _L_, left; LG, lingual gyrus; m, medial; MF, medial frontal cortex; MidB, midbrain; p, posterior; Par, parahippocampal gyrus; PoCG, postcentral gyrus; PrCG, precentral gyrus; _R_, right; s, superior; SMG, supramarginal gyrus; TG, temporal gyrus; TOF, temporal occipital fusiform gyrus; TP, temporal gyrus. Where no laterality is noted (_L_ or _R_), the cluster was represented bilaterally. Where there are two data sets for one brain region, there were two clusters in that region

Regional brain responses to hypoglycaemia in IAH involved various regions similar to those showing responses in the HA group (Fig. 2, ESM Tables 5–7), with some differences that were explored in a formal comparison. Directly comparing responses between groups identified clusters showing significantly different hypoglycaemia responses between the IAH and HA groups at the second statistical threshold voxel level (*p* < 0.005, *k* ≥ 50 voxels) (Fig. 3; Table 1). In early hypoglycaemia (Fig. 3a–c), five clusters were identified. One in the left lingual gyrus showed deactivation for IAH and activation for HA, and four—the right central operculum, medial orbital (MO) cortex bilaterally and left posterior DLF cortex (two clusters)—showed activation in IAH vs deactivation in HA.

**Table 1:** Regions of the brain with different activation responses to hypoglycaemia in men with type 1 diabetes with HA vs those with IAH

Period vs baseline, response direction	Group (region name)	*k* (voxels)	*t* (peak voxel)	*p*(peak voxel)	*p*(uncorrected cluster)	Atlas coordinates for location in the brain			Subcluster size
						x	y	z	
Early									
HA > IAH	Lingual gyrus L	54	3.57	0.00024	0.060	0	−74	−6	31
	Lingual gyrus R								23
HA < IAH	Post dorsolateral frontal cortex L	79	−3.47	0.00034	0.026	−48	22	12	79
	Post dorsolateral frontal cortex L	54	−4.40	0.00001	0.060	−32	26	46	54
	Insular cortex R^a^	55	−3.53	0.00027	0.058	44	−2	10	4
	Precentral gyrus R^a^								3
	Central operculum cortex R								44
	Cerebral white matter R^a^								4
	Medial orbital cortex L^a^	70	−4.17	0.00003	0.035	4	62	−14	11
	Medial orbital cortex R								59
Established									
HA > IAH	Precentral gyrus L^a^	128	3.84	0.00009	0.007	−6	−38	66	9
	Postcentral gyrus L								95
	Cerebral white matter R^a^								30
	Precentral gyrus R	89	4.01	0.00005	0.020	20	−28	66	56
	Postcentral gyrus R								33
	Dorsal ant cingulate cortex								42
	Cerebral white matter R^a^								8
	Ant cingulate cortex	68	4.03	0.00004	0.037	−2	36	6	68
HA < IAH	Parietal lobule L	109	−4.75	<0.00001	0.011	−34	−58	40	54
	Supramarginal gyrus L^a^								2
	Angular gyrus L								53
	Middle temp gyrus post L	171	−4.18	0.00002	0.002	−54	−62	16	76
	Angular gyrus L								63
	Occipital pole L								32
	Medial orbital cortex L^a^	133	−3.76	0.00012	0.006	4	60	−12	6
	Medial orbital cortex R								127
Recovery									
HA > IAH	Outside atlas^a^	127	3.53	0.00027	0.007	40	−40	−16	4
	Inf temp gyrus post R^a^								1
	Cerebral white matter R^a^								7
	Postcentral gyrus L								58
	Outside atlas^a^	111	3.79	0.00011	0.010	10	−8	−16	23
	Cerebral white matter R^a^								17
	Hippocampus R^a^								9
	Amygdala R								22
	Midbrain								30
	Precentral gyrus L	119	3.84	0.00009	0.008	−38	−24	62	25
	Postcentral gyrus L								94
	Insular cortex R	78	3.42	0.0004	0.027	42	−22	0	11
	Sup temp gyrus ant R								52
	Sup temp gyrus post R^a^								7
	Cerebral white matter R^a^								8
	Outside atlas^a^	79	3.81	0.0001	0.026	32	2	−20	3
	Insular cortex R^a^								10
	Ant temp pole R								21
	Parahippocampal ambiens gyrus ant R^a^								1
	Cerebral white matter R								27
	Amygdala R								17
HA < IAH	Middle temp gyrus ant L	97	−5.02	<0.00001	0.015	−64	−14	−14	32
	Middle temp gyrus post L								56
	Inf temp gyrus ant L^a^								3
	Inf temp gyrus post L^a^								6
	Sup temp gyrus post L	138	−4.56	<0.00001	0.005	−58	−48	16	43
	Middle temp gyrus post L^a^								1
	Supramarginal gyrus L								68
	Angular gyrus L								23
	Cerebral white matter L^a^								3
	Ant dorsolateral frontal cortex L^a^	78	−4.42	<0.00001	0.027	−10	50	34	10
	Anterior medial frontal cortex L^a^								6
	Postero-medial frontal cortex L								62
	Middle temp gyrus post L	90	−4.01	0.00005	0.019	−56	−34	−22	22
	Inf temp gyrus post L								68
	Parietal lobule L	685	−3.96	0.00006	<0.001	−38	−58	54	88
	Supramarginal gyrus L								38
	Angular gyrus L								425
	Occipital pole L								134
	Middle temp gyrus post R	53	−3.89	0.00007	0.062	60	−46	2	53
	Precentral gyrus L	95	−3.57	0.00024	0.016	−40	2	52	32
	Post dorsolateral frontal cortex L								63
	Post dorsolateral frontal cortex L	97	−3.46	0.00035	0.015	−36	18	50	97

Region names derive from the intersection of regions identified by SPM using the Tziortzi atlas [29]. *t* values are calculated from a linear contrast for every voxel, the peak *t* value being the voxel with highest *t* value in the cluster

*p*(peak voxel) is the *p* value that corresponds to the peak *t* value within each cluster, calculated with 154 degrees of freedom. *p*(uncorrected cluster) is the *p* value calculated using SPM2, reflecting the significance of cluster size of regions that meet the criteria for significance (i.e. the likelihood that a cluster of that size being found based on Gaussian field theory and functional image smoothness)

^a^Regions that did not meet the criteria for significance

Ant, anterior; Inf, inferior; L left; Post, posterior; R right; Sup, superior; Temp, temporal

As hypoglycaemia continued (Fig. 3d–f), eight clusters showed significantly different changes in activation between groups. Five showed limited or absent responses for IAH but activation for HA: the right GP, ACC, dorsal cingulate cortex, right pre- and bilateral post-central gyri, and left precuneus. Two further clusters, in the right MO and left parietal cortices (parietal lobule and angular gyrus), showed activation in IAH but no change or deactivation in HA. A cluster including the left posterior middle temporal gyrus, angular gyrus and occipital pole showed no change in IAH but deactivation in HA. Fourteen clusters showed significantly different activation between groups during the recovery period (Fig. 3g–i). IAH showed deactivation or minimal response compared with activation in the HA group in the following: right fusiform cortex; a midbrain cluster, extending to the right amygdala; right superior temporal gyrus/insula; left pre- and post-central gyri; and a cluster including right white matter, anterior temporal pole and amygdala. The IAH group failed to show the deactivation seen with HA in the bilateral middle and left inferior temporal gyri. However, there was activation in IAH compared with deactivation in HA in the left posterior medial frontal cortex, left posterior DLF cortex extending to pre-central gyrus, and left angular gyrus/occipital pole/parietal lobule/supramarginal gyrus/left posterior superior temporal gyrus.

## Discussion

In this neuroimaging study of men with type 1 diabetes, differences in symptomatic and hormonal responses to hypoglycaemia between participants with preserved (HA) or impaired (IAH) awareness of hypoglycaemia were accompanied by subtle differences in brain responses. These occurred not only in regions associated with the generation and subjective awareness of stress responses, but also in regions associated with executive control, reward, memory and emotional salience.

Defective hormonal responses to hypoglycaemia in IAH are well recognised [2, 8, 16]. They are inducible by exposure to hypoglycaemia [7, 8] and restored by avoiding it [2, 20]. The IAH group showed the expected diminution of symptomatic and catecholamine responses to our hypoglycaemic challenge.

### Responses in men with type 1 diabetes and intact awareness

Our neuroimaging data in HA participants are largely consistent with studies in people without diabetes and extend earlier observations, particularly by describing the evolution of responses during development of and recovery from hypoglycaemia. Our ‘early’ scans were made as arterialised plasma glucose level was falling, and include data collected at glucose values between 3.5 and 2.2 mmol/l; the ‘established’ data were collected at 2.4 mmol/l. Thalamic activation seen in both early and established hypoglycaemia is consistent with data from individuals without diabetes, and with the role of the thalamus in relaying sensory signals to cortical areas [30]. Novel findings include insula activation, seen in all three phases of hypoglycaemia and not previously described in diabetes. Using similar techniques, we previously described insular activation in established hypoglycaemia only in people without diabetes [14]; we speculate that earlier and more persistent activation in type 1 diabetes relates to heightened sensitivity to changes in plasma glucose or prior experience of more fluctuating glucose concentrations. Wiegers et al did not find insular activation in people without diabetes and described reduced insular perfusion using functional MRI (fMRI) in seven HA individuals with type 1 diabetes [19]. Comparing direction of signal change across studies using different technologies is complex; however, [^15^O]water PET is less susceptible to low signal to noise ratios and movement than fMRI. It also allows more quantitative measurement of regional responses and may be better at detecting differences between groups in studies of similar size [31].

Activation of the GP in established hypoglycaemia and recovery, and the ACC in all three phases, is consistent with some reports involving individuals without diabetes at similar glucose concentrations [13, 14] but has not been described in type 1 diabetes. The GP and ACC, involved in reward, might be reacting to the hypoglycaemic stress responses. Activation of orbital cortex is likewise consistent with non-diabetic responses to comparable hypoglycaemia [13, 14, 18] but not previously observed in type 1 diabetes [19]. The orbital cortex encodes stimulus value or salience [32], and the lateral orbital cortex forms a ‘salience’ network with the ACC [33]. Activation of the DLF cortex in response to hypoglycaemia has not previously been described [12–14, 18, 19]. It has a role in working memory—the short-term recall and processing of information necessary for complex task performance, including learning and reasoning [34]—and is involved in inhibitory control [35, 36]. Changing activity during hypoglycaemia is consistent with clinically observed changes in cognition and behavioural disinhibition during hypoglycaemia, and recall after it. The report of activation of the precuneus and angular gyrus during established hypoglycaemia and recovery is also novel [12–14, 19]. The precuneus is part of the ‘default mode network’, showing reduced activity compared with the resting state when undertaking tasks [33]; activation may reflect lesser ability to perform tasks during hypoglycaemia. The angular gyrus, linked to the DLF cortex [37] and showing parallel responses, plays a role in regulating shift of attention to more salient stimuli [38]. The amygdala encodes the predicted biological relevance of a stimulus [39]; its activation in recovery may be a key determinant of responses to subsequent hypoglycaemic events.

Deactivation of the inferior temporal gyri in all three phases, of parietal regions during established hypoglycaemia and recovery, and of parahippocampal regions during established hypoglycaemia, described in some studies of individuals without diabetes but not previously in type 1 diabetes, provide a neurological correlate of failure to form memory during hypoglycaemia: temporal gyri for semantic or conceptual memory [40], and parahippocampal gyrus and lateral parietal cortex for episodic memory [41].

### Impact of IAH

The subtle differences in hypoglycaemia responses in IAH are potentially important. In early hypoglycaemia, deactivation seen in parts of the central operculum, MO cortex and posterior and lateral DLF cortex in HA was replaced by activation. Operculum activation changes in response to food cues, modulated by feeding state and degree of liking the food [32]: differences between IAH and HA may relate to differences in the drive to eat to treat. These were paralleled by different responses in the MO cortex, encoding stimulus value and salience [32], in early and established hypoglycaemia; this may underlie differences in perceived importance of hypoglycaemia, including lack of aversion. Lack of activation of the GP, with its role in memory of unpleasant experiences or aversion [42], is also consistent with not finding hypoglycaemia unpleasant. These are key findings, as IAH is clinically associated with reduced motivation to avoid hypoglycaemia [21, 22], with reduced incentive to treat hypoglycaemia as important [43].

Parts of the dorsal and posterior DLF cortex responded from early hypoglycaemia through to recovery with activation responses in IAH compared with deactivation in HA. Hypoglycaemia is associated with impaired inhibitory control; perhaps the deactivation in HA represents conscious attempts to maintain inhibitory control of behaviour during hypoglycaemia.

The ACC is involved in decision-making and conflict resolution between options, and is key in monitoring performance, evaluating actions and detecting events that require behavioural modification and re-evaluation [44]. Lack of ACC activation only in IAH fits with views of IAH as a habituation response. A similar lack of activation in IAH in the somatosensory post-central (somatosensory) and pre-central (motor) gyri, persisting in recovery, may reflect reduced somatic sensations (e.g. warmth, shakiness) and motor responses (e.g. tremor) experienced by the IAH group in hypoglycaemia.

Minimal responses in IAH, vs deactivation in HA, in the left posterior middle temporal gyrus during established hypoglycaemia, and the bilateral posterior middle and left inferior temporal gyri in recovery, are also consistent with different memory formation during hypoglycaemia and recovery [40, 41]. The same is true of differences in the left parietal lobule/angular gyrus in established hypoglycaemia, with activation in IAH but minimal response in HA, and in recovery in the left angular gyrus and supramarginal gyrus, with activation in IAH and deactivation in HA. The lateral parietal cortex shows functional connectivity with the hippocampal formation and is associated with recollection of experiences [41, 45].

In recovery, in addition to persisting differential responses in somatosensory and memory networks, we found activation in IAH and deactivation in HA in part of the medial frontal cortex; this was in a cluster corresponding to regions of the dorsal-medial prefrontal cortex identified as having a role in self-referential mental activity, such as making judgements about unpleasantness/pleasantness [46]. It may also have a role in episodic or experiential memory [41]. This may provide a correlate for individuals with type 1 diabetes with IAH and HA forming differently valenced memories of the experience of hypoglycaemia. However, the medial frontal cortex, along with the lateral parietal regions discussed above, is also a component of the default mode network [33], and these differences may represent hypoglycaemia being a different ‘task’ for the brain in IAH than HA.

Our participants were matched well for age, diabetes duration and diabetes control but imperfectly for BMI. Obesity alters brain responses to food and food cues, including the responses of some frontal regions described as different in their response to hypoglycaemia here [47]. However, none of our participants was obese, so it is unlikely our observed differences in response to hypoglycaemia were related to this. The recruitment of only men facilitates research involving radio-isotopes and importantly reduces variability of responses due to sex differences in counterregulation [48]. The clinical picture of IAH is not sex-specific so our data interpretation probably also applies to women; however, adaptation to antecedent hypoglycaemia may vary by sex, at least in individuals without diabetes [49], and studies in women would be of interest. Right-handedness was chosen as many brain functions are lateralised.

The strengths of our study include pre-study determination of awareness status on clinical grounds, so individuals defined as having IAH were representative of those with clinically problematic hypoglycaemia. In addition, the analysis did not require a preconception of brain regions that might respond differently to hypoglycaemia by awareness status. Although less powerful than a region of interest analysis, in which data are compared between groups only in prespecified brain regions, this enabled us to identify areas not traditionally associated with stress responses. That differences between the two groups (the effect of awareness status on rCBF responses) were identified at lower thresholds than those used to find differences within groups (the effect of hypoglycaemia) is statistically explicable: within-group comparison is always more powerful than between-group comparison, where differences between participants come into play. It is also biologically plausible as hypoglycaemia is a large stress stimulus whereas differences between HA and IAH are probably an order of magnitude less. It is, however, possible that other brain regions responding differently were missed.

## Conclusion

In conclusion, we used [^15^O]water PET to describe the evolution of the brain’s responses to hypoglycaemia over time in men with type 1 diabetes and found differences related to HA status. These differences provide a mechanism explaining the resilience of IAH as a clinical entity highly resistant to treatment strategies that are usually capable of restoring awareness through hypoglycaemia avoidance. The neuroimaging differences are compatible with a different behavioural response, with regard to the drive to eat, different emotional salience of the experience and differences in its recall; all may contribute in IAH to the reduced drive to treat hypoglycaemia in timely fashion and avoid future episodes. It remains to be determined whether these IAH-specific central responses are induced by hypoglycaemia exposure or are an inherent way of responding to stress that results in a proportion of people susceptible to persistent IAH. If the latter, it may be possible to detect high risk for IAH and recurrent severe hypoglycaemia through cognitive or neuroimaging studies before the syndrome has fully developed. Meanwhile, the differential responses described are likely to correlate with cognitions and behaviours unhelpful to future hypoglycaemia avoidance; further research is required into how best to address these in clinical practice.

## Electronic supplementary material

ESM (PDF 1.45 mb)

## Data Availability

The primary data are medical imaging data for which there are no publicly available repositories. The authors are able to provide data in response to email requests.

## Abbreviations

ACC: Anterior cingulate cortex
CT: Computerised tomography
DLF: Dorsolateral frontal
fMRI: Functional MRI
GP: Globus pallidus
HA: Hypoglycaemia awareness
IAH: Impaired awareness of hypoglycaemia
iAUC: Incremental AUC
*k*: Cluster size
MO: Medial orbital
PET: Positron emission tomography
rCBF: Regional cerebral blood flow
SPM: Statistical Parametric Mapping 2
