# Supplementary material for: The impact of hypoglycaemia awareness status on regional brain responses to acute hypoglycaemia in men with type 1 diabetes
Source: Diabetologia. 2018 May 12;61(7):1676–87. doi: 10.1007/s00125-018-4622-2 (PMC6445483; doi:10.1007/s00125-018-4622-2)
Supplement: Supplementary file 1 — (PDF 1.45 mb) [file 125_2018_4622_MOESM1_ESM.pdf]

ESM Table 1: Mean & S.D of intergral Area Under the Curve (iAUC) of change in hormone, catecholamine and symptom scores from baseline.

	HA		IAH		p-value
	Mean	SD	Mean	SD	*group comparison*
Adrenaline	110.9	52.4	45.7	28.5	0.007
p-value (iAUC > 0)	*<0.001*		*0.001*		
Noradrenaline	23.10	20.35	6.70	18.23	NS
p-value (iAUC > 0)	*0.005*		*0.166*		
Cortisol	6740	10810	-116	4162	NS
p-value (iAUC > 0)	*0.075*		*0.528*		
Growth Hormone	759.0	911.6	845.1	632.2	NS
p-value (iAUC > 0)	*0.035*		*0.006*		
Total Autonomic	422.7	237.1	26.2	35.5	0.002
p-value (iAUC > 0)	*0.001*		*0.038*		
Total Neuroglycopenic	478.9	311.1	34.8	88.8	0.002
p-value (iAUC > 0)	*0.002*		*0.152*		

NS: no significant interaction effect in mixed model

ESM Table 2: All brain regions with change in activity during early hypoglycaemia vs baseline in subjects with intact hypoglycaemia awareness (HA).

Period compared to baseline	Response direction	Group (Region Name)	Cluster Size *k* (voxels)	T (peak)	P (corrected cluster)	coordinates			Subcluster Size
						x	y	z	
**EARLY**									
	**Increase**	**HA**							
		Ant_Dorsolateral_Frontal_cortex_R	697	6.49	<0.001	26	60	2	566
		Medial_Orbital_cortex_R							31
		Lat_Orbital_cortex_R							100
		outside atlas[a]	443	5.21	<0.001	42	26	-4	8
		Insular_cortex_R[a]							5
		Anterior_Temp_Pole_R							20
		Lat_Orbital_cortex_R							354
		Frontal_Operculum_cortex_R							53
		Post_Dorsolateral_Frontal_cortex_R[a]							3
		Cerebral_White_Matter_R[a]	141	3.83	0.008	8	-24	6	1
		Th_Primary_Sensory_R[a]							1
		Th_Pre_frontal_R							82
		Th_Pre_motor_R[a]							1
		Th_Posterior_Parietal_R							19
		Th_Temp_R							37
		outside atlas[a]	106	5.00	0.032	-14	-30	2	4
		Cerebral_White_Matter_L[a]							1
		Th_Occipital_L[a]							6
		Th_Pre_frontal_L							13
		Th_Posterior_Parietal_L							51

		Th_Temp_L							31
		Ant_Dorsolateral_Frontal_cortex_L	497	4.98	<0.001	-32	52	8	490
		Anterior_Medial_Frontal_cortex_L[a]							5
		Poster_Medial_Frontal_cortex_L[a]							2
		Ant_Dorsolateral_Frontal_cortex_R[a]	114	5.00	0.023	30	28	46	11
		Post_Dorsolateral_Frontal_cortex_R							103
		Supramarginal_gyrus_R	116	4.47	0.022	50	-48	50	25
		Angular_gyrus_R							91
		Ant_Dorsolateral_Frontal_cortex_R	131	4.19	0.012	40	38	28	131
		Anterior_Cingulate_G	250	4.71	<0.001	2	50	6	242
		Anterior_Medial_Frontal_cortex_R[a]							7
		Anterior_Medial_Frontal_cortex_L[a]							1
	**Decrease**	**HA**							
		Inf_Temp_gyrus_post_L	107	-4.29	0.031	-50	-44	-22	106
		Temp_Fusiform_cortex_post_L[a]							1
		Dorsal_Cerebellum_L[a]	176	-4.15	0.002	-44	-46	-36	17
		Ventrolateral_Cerebellum_L							159

[a] Regions that did not meet criteria for significance

ESM Table 3: All brain regions with change in activity during established hypoglycaemia vs baseline in subjects with intact hypoglycaemia awareness (HA)

Period compared to baseline	Response direction	Group (Region Name)	Cluster Size, *k* (voxels)	T (peak)	P (corrected cluster)	coordinates			Subcluster Size
						x	y	z	
**ESTABLISHED**									
	**Increase**	**HA**							
		outside atlas[a]	3662	8.59	<0.001	14	-24	6	311
		Parahip_Subiculum_gyrus_post_L[a]							9
		Parahip_Subiculum_gyrus_post_R[a]							5
		Posterior_Cingulate_cortex[a]							6
		Cerebral_White_Matter_L							268
		Globus_Pallidus_L							260
		Hippocampus_L[a]							10
		Accumbens_L[a]							6
		Cerebral_White_Matter_R							241
		Globus_Pallidus_R							233
		Hippocampus_R[a]							10
		Th_Primary_Motor_L							43
		Th_Primary_Sensory_L[a]							17
		Th_Occipital_L							24
		Th_Pre_frontal_L							477
		Th_Pre_motor_L							84
		Th_Posterior_Parietal_L							196
		Th_Temp_L							149
		Th_Primary_Motor_R[a]							11
		Th_Primary_Sensory_R							39
		Th_Occipital_R[a]							2
		Th_Pre_frontal_R							402
		Th_Pre_motor_R							89

		Th_Posterior_Parietal_R							204
		Th_Temp_R							201
		Midbrain							245
		PreDorsalPutamen_R[a]							1
		PreVentralPutamen_R[a]							8
		PosDorsalPutamen_R							60
		PosVentralPutamen_R[a]							12
		PreDorsalPutamen_L[a]							1
		PreVentralPutamen_L[a]							7
		PosDorsalPutamen_L							22
		PosVentralPutamen_L[a]							9
		Ant_Dorsolateral_Frontal_cortex_R	2766	6.81	<0.001	26	60	0	1699
		Precentral_gyrus_R[a]							9
		Medial_Orbital_cortex_R[a]							4
		Lat_Orbital_cortex_R							277
		Anterior_Medial_Frontal_cortex_R							70
		Anterior_Medial_Frontal_cortex_L							34
		Post_Dorsolateral_Frontal_cortex_R							673
		outside atlas[a]	837	6.28	<0.001	42	28	-2	16
		Insular_cortex_R							178
		Ant_Dorsolateral_Frontal_cortex_R[a]							2
		Anterior_Temp_Pole_R							40
		Lat_Orbital_cortex_R							407
		Frontal_Operculum_cortex_R							136
		Cerebral_White_Matter_R[a]							1
		Post_Dorsolateral_Frontal_cortex_R							57
		outside atlas[a]	2822	7.26	<0.001	-28	52	10	1
		Insular_cortex_L							85

		Ant_Dorsolateral_Frontal_cortex_L							1892
		Precentral_gyrus_L							49
		Medial_Orbital_cortex_L							31
		Lat_Orbital_cortex_L							199
		Frontal_Operculum_cortex_L							54
		Anterior_Medial_Frontal_cortex_L[a]							14
		Post_Dorsolateral_Frontal_cortex_L							497
		Anterior_Cingulate_cortex	909	6.17	<0.001	-2	36	6	692
		Dorsal_Anterior_Cingulate							175
		Cerebral_White_Matter_R[a]							12
		Poster_Medial_Frontal_cortex_R[a]							12
		Poster_Medial_Frontal_cortex_L[a]							18
		outside atlas[a]	131	5.07	0.012	-48	-28	6	1
		Sup_Temp_gyrus_ant_L							62
		Sup_Temp_gyrus_post_L							66
		Central_Operculum_cortex_L[a]							2
		Precentral_gyrus_R	143	4.62	0.008	46	0	32	73
		Postcentral_gyrus_R							70
		Parietal_Lobule_R[a]	179	4.52	0.002	46	-48	44	3
		Supramarginal_gyrus_R							79
		Angular_gyrus_R							97
		Precuneous_cortex_L[a]	111	4.32	0.026	8	-80	46	9
		Parietal_Lobule_R[a]							10
		Occipital_Pole_R[a]							7
		Precuneous_cortex_R							83
		Cuneus_R[a]							2

		outside atlas[a]	1169	5.91	<0.001	42	-26	64	1
		Precentral_gyrus_L[a]							10
		Postcentral_gyrus_L							186
		Precuneous_cortex_L[a]							74
		Precentral_gyrus_R							108
		Postcentral_gyrus_R							622
		Parietal_Lobule_R							74
		Supramarginal_gyrus_R[a]							8
		Precuneous_cortex_R							86
		Parietal_Lobule_R	103	4.44	0.036	26	-68	54	102
		Angular_gyrus_R[a]							1
		Sup_Temp_gyrus_ant_R[a]	111	5.37	0.026	56	-18	4	12
		Sup_Temp_gyrus_post_R							98
		Cerebral_White_Matter_R[a]							1
	**Decrease**	**HA**							
		Anterior_Temp_Pole_R	224	-4.59	<0.001	38	-10	-42	95
		Inf_Temp_gyrus_ant_R							20
		Inf_Temp_gyrus_post_R[a]							5
		Temp_Fusiform_cortex_ant_R							80
		Temp_Fusiform_cortex_post_R							24
		outside atlas[a]	3455	-6.93	<0.001	-50	-44	-24	38
		Anterior_Temp_Pole_L							335
		Sup_Temp_gyrus_ant_L							111
		Sup_Temp_gyrus_post_L[a]							3
		Middle_Temp_gyrus_ant_L							137
		Middle_Temp_gyrus_post_L							247

		Inf_Temp_gyrus_ant_L							99
		Inf_Temp_gyrus_post_L							737
		Supramarginal_gyrus_L[a]							13
		Angular_gyrus_L							116
		Occipital_Pole_L							162
		Parahip_Ambiens_gyrus_ant_L[a]							17
		Parahip_Subiculum_gyrus_post_L							78
		Lingual_gyrus_L							59
		Temp_Fusiform_cortex_ant_L							147
		Temp_Fusiform_cortex_post_L							360
		Temp_Occip_Fusiform_cortex_L							142
		Occipital_Fusiform_gyrus_L[a]							3
		Posterior_Cingulate_cortex[a]							1
		Cerebral_White_Matter_L							38
		Hippocampus_L							190
		Dorsal_Cerebellum_L							362
		Ventrolateral_Cerebellum_L							60
		outside atlas[a]	110	-7.08	0.027	30	-42	-2	2
		Lingual_gyrus_R							32
		Temp_Occip_Fusiform_cortex_R							20
		Posterior_Cingulate_cortex[a]							1
		Cerebral_White_Matter_R[a]							31
		Lat_Ventricle_R[a]							1
		Hippocampus_R							23
		outside atlas	1779	-5.41	<0.001	26	-36	-26	30
		Anterior_Temp_Pole_R							177
		Sup_Temp_gyrus_ant_R							30
		Middle_Temp_gyrus_ant_R							168
		Middle_Temp_gyrus_post_R							212

		Inf_Temp_gyrus_ant_R							26
		Inf_Temp_gyrus_post_R							376
		Angular_gyrus_R							68
		Occipital_Pole_R							136
		Parahip_Subiculum_gyrus_post_R[a]							10
		Lingual_gyrus_R[a]							8
		Temp_Fusiform_cortex_post_R							124
		Temp_Occip_Fusiform_cortex_R							66
		Dorsal_Cerebellum_R							312
		Ventrolateral_Cerebellum_R							36
		outside atlas[a]	272	-5.23	<0.001	22	-72	-22	1
		Dorsal_Cerebellum_R							204
		Ventrolateral_Cerebellum_R							67
		Cerebral_White_Matter_R[a]	126	-4.90	0.015	32	-18	-14	54
		Hippocampus_R							72
		outside atlas[a]	2477	-5.90	<0.001	2	-60	-22	16
		Temp_Occip_Fusiform_cortex_L[a]							5
		Dorsal_Cerebellum_R							89
		Dorsal_Cerebellum_L							248
		Ventrolateral_Cerebellum_R							431
		Ventrolateral_Cerebellum_L							914
		Medial_Cerebellum_R							192
		Medial_Cerebellum_L							553
		Pons[a]							14
		Medulla[a]							15

[a] Regions that did not meet criteria for significance

ESM Table 4 lists all brain regions with change in activity during recovery from hypoglycaemia vs baseline in subjects with intact hypoglycaemia awareness (HA)

Period compared to baseline	Response direction	Group (Region Name)	Cluster Size, *k* (voxels)	T (peak)	P (corrected cluster)	coordinates			Subcluster Size
						x	y	z	
**RECOVERY**									
	**Increase**	**HA**							
		Cerebral_White_Matter_L[a]	134	4.54	0.011	-24	-12	-10	53
		Globus_Pallidus_L							54
		Hippocampus_L[a]							1
		Amygdala_L							26
		outside atlas[a]	116	4.29	0.022	-34	-26	6	4
		Insular_cortex_L[a]							12
		Sup_Temp_gyrus_ant_L							23
		Sup_Temp_gyrus_post_L[a]							7
		Parietal_Operculum_cortex_L[a]							4
		Cerebral_White_Matter_L[a]							49
		Th_Posterior_Parietal_L[a]							17
		Anterior_Cingulate_cortex	389	5.58	<0.001	-4	38	8	368
		Dorsal_Anterior_Cingulate cortex[a]							3
		Cerebral_White_Matter_L[a]							1
		Cerebral_White_Matter_R[a]							1
		Anterior_Medial_Frontal_cortex_L[a]							16
		outside atlas[a]	260	4.67	<0.001	18	-2	-4	6
		Insular_cortex_R[a]							19
		Cerebral_White_Matter_R							43
		Globus_Pallidus_R							109

		Hippocampus_R[a]							5
		Amygdala_R[a]							19
		Midbrain							40
		PreVentralPutamen_R[a]							2
		PosDorsalPutamen_R[a]							17
		Precentral_gyrus_L[a]	110	5.17	0.027	-42	-28	48	12
		Postcentral_gyrus_L							98
		outside atlas[a]	100	4.48	0.041	-24	10	-14	1
		Insular_cortex_L[a]							1
		Lat_Orbital_cortex_L							29
		Ventral_Cing_Subcallosal_G							26
		Cerebral_White_Matter_L							40
		PreVentralPutamen_L[a]							3
	**Decrease**	**HA**							
		outside atlas[a]	1103	-6.42	<0.001	-52	-44	-24	3
		Sup_Temp_gyrus_post_L[a]							2
		Middle_Temp_gyrus_ant_L							49
		Middle_Temp_gyrus_post_L							296
		Inf_Temp_gyrus_ant_L[a]							19
		Inf_Temp_gyrus_post_L							631
		Occipital_Pole_L							45
		Parahip_Ambiens_gyrus_ant_L[a]							2
		Temp_Fusiform_cortex_post_L							32
		Temp_Occip_Fusiform_cortex_L[a]							9
		Hippocampus_L[a]							3
		Dorsal_Cerebellum_L[a]							12
		outside atlas[a]	231	-4.81	<0.001	-38	-72	-38	1

		Temp_Occip_Fusiform_cortex_L[a]							2
		Occipital_Fusiform_gyrus_L[a]							8
		Dorsal_Cerebellum_L							86
		Ventrolateral_Cerebellum_L							134
		Middle_Temp_gyrus_post_R	970	-5.01	<0.001	56	-40	-22	439
		Inf_Temp_gyrus_ant_R							21
		Inf_Temp_gyrus_post_R							401
		Supramarginal_gyrus_R[a]							9
		Angular_gyrus_R							66
		Occipital_Pole_R							34
		Parietal_Lobule_L[a]	127	-4.31	0.014	-36	-78	34	10
		Angular_gyrus_L							22
		Occipital_Pole_L							95
		Ventrolateral_Cerebellum_L	135	-4.91	0.010	-40	-46	-42	135
		Dorsal_Cerebellum_L	233	-4.62	<0.001	-16	-74	-34	33
		Ventrolateral_Cerebellum_L							174
		Medial_Cerebellum_L							26
		Dorsal_Cerebellum_R	116	-3.90	0.022	24	-72	-34	59
		Ventrolateral_Cerebellum_R							56
		Medial_Cerebellum_R[a]							1
		Angular_gyrus_R	136	-4.00	0.010	50	-60	28	91
		Occipital_Pole_R							45
		Supramarginal_gyrus_L	420	-4.88	<0.001	-52	-54	40	25
		Angular_gyrus_L							334

		Occipital_Pole_L							61

[a] Regions that did not meet criteria for significance

ESM Table 5: All brain regions with change in activity during early hypoglycaemia vs baseline in subjects with impaired awareness of hypoglycaemia (IAH)

Period compared to baseline	Response direction	Group (Region Name)	Cluster Size, *k* (voxels)	T (peak)	P (corrected cluster)	coordinates			Subcluster Size
						x	y	z	
**EARLY**									
	**Increase**	**IAH**							
		Ant_Dorsolateral_Frontal_cortex_L	349	4.57	<0.001	-42	42	-4	329
		Lat_Orbital_cortex_L[a]							18
		Cerebral_White_Matter_L[a]							2
		outside atlas[a]	162	5.10	0.004	48	24	-12	8
		Insular_cortex_R[a]							11
		Anterior_Temp_Pole_R[a]							4
		Lat_Orbital_cortex_R							101
		Frontal_Operculum_cortex_R							10
		Post_Dorsolateral_Frontal_cortex_R							28
		outside atlas[a]	281	7.09	<0.001	14	-28	10	2
		Cerebral_White_Matter_R[a]							1
		Th_Pre_frontal_L							68
		Th_Temp_L							46
		Th_Pre_frontal_R							38
		Th_Posterior_Parietal_R							49
		Th_Temp_R							77
		outside atlas[a]	176	4.58	0.002	-38	20	-8	3
		Insular_cortex_L							22
		Lat_Orbital_cortex_L							90
		Frontal_Operculum_cortex_L							21

		Post_Dorsolateral_Frontal_cortex_L							40
	**Decrease**	**IAH**							
		outside atlas[a]	105	-4.11	0.033	48	-52	-20	6
		Inf_Temp_gyrus_post_R							28
		Temp_Occip_Fusiform_cortex_R							38
		Dorsal_Cerebellum_R							29
		Ventrolateral_Cerebellum_R[a]							4
		outside atlas[a]	100	-4.05	0.041	-42	-54	-30	2
		Temp_Fusiform_cortex_post_L							37
		Dorsal_Cerebellum_L							58
		Ventrolateral_Cerebellum_L[a]							3

ESM Table 6: All brain regions with change in activity during established hypoglycaemia vs baseline in subjects with impaired awareness of hypoglycaemia (IAH)

Period compared to baseline	Response direction	Group (Region Name)	Cluster Size, *k* (voxels)	T (peak)	P (corrected cluster)	coordinates			Subcluster Size
						x	y	z	
ESTABLISHED									
	**Increase**	**IAH**							
		outside atlas	2873	9.92	<0.001	14	-28	8	265
		Parahip_Subiculum_gyrus_post_L[a]							15
		Parahip_Subiculum_gyrus_post_R							28
		Lingual_gyrus_R[a]							1
		Posterior_Cingulate_G[a]							8
		Cerebral_White_Matter_L[a]							108
		Globus_Pallidus_L							135
		Hippocampus_L							33
		Cerebral_White_Matter_R							150
		PreCaudate_R[a]							1
		Globus_Pallidus_R							119
		Hippocampus_R							26
		Th_Primary_Motor_L[a]							15
		Th_Primary_Sensory_L[a]							5
		Th_Occipital_L[a]							17
		Th_Pre_frontal_L							395
		Th_Pre_motor_L							48
		Th_Posterior_Parietal_L							126
		Th_Temp_L							205
		Th_Primary_Motor_R[a]							9
		Th_Primary_Sensory_R							37
		Th_Occipital_R[a]							5
		Th_Pre_frontal_R							390

		Th_Pre_motor_R							54
		Th_Posterior_Parietal_R							202
		Th_Temp_R							245
		Midbrain							175
		PreDorsalPutamen_R[a]							3
		PreVentralPutamen_R[a]							2
		PosDorsalPutamen_R							31
		PosDorsalPutamen_L[a]							2
		PosVentralPutamen_L							18
		outside atlas[a]	583	6.75	<0.001	-34	-58	40	3
		Parietal_Lobule_L							243
		Supramarginal_gyrus_L[a]							4
		Angular_gyrus_L							246
		Occipital_Pole_L							87
		outside atlas[a]	3207	6.67	<0.001	-34	52	-2	3
		Insular_cortex_L							85
		Ant_Dorsolateral_Frontal_cortex_L							1376
		Precentral_gyrus_L							170
		Medial_Orbital_cortex_L							91
		Lat_Orbital_cortex_L							392
		Frontal_Operculum_cortex_L							30
		Cerebral_White_Matter_L[a]							4
		Anterior_Medial_Frontal_cortex_L							154
		Post_Dorsolateral_Frontal_cortex_L							902
		outside atlas[a]	3226	6.41	<0.001	52	26	-6	9
		Insular_cortex_R							69
		Ant_Dorsolateral_Frontal_cortex_R							1381
		Precentral_gyrus_R							157

		Anterior_Temp_Pole_R[a]							17
		Postcentral_gyrus_R[a]							4
		Medial_Orbital_cortex_R							107
		Lat_Orbital_cortex_R							724
		Frontal_Operculum_cortex_R							35
		Cerebral_White_Matter_R[a]							1
		Anterior_Medial_Frontal_cortex_R							22
		Post_Dorsolateral_Frontal_cortex_R							700
		outside atlas[a]	384	4.83	<0.001	0	28	44	2
		SMA_L[a]							11
		Anterior_Cingulate_cortex[a]							7
		Dorsal_Anterior_Cingulate_cortex							115
		Cerebral_White_Matter_L[a]							5
		Poster_Medial_Frontal_cortex_R							124
		Poster_Medial_Frontal_cortex_L							120
		Supramarginal_gyrus_R	150	4.65	0.006	48	-46	46	58
		Angular_gyrus_R							92
		Ant_Dorsolateral_Frontal_cortex_R	144	4.36	0.007	24	28	52	39
		Post_Dorsolateral_Frontal_cortex_R							105
	**Decrease**	**IAH**							
		outside atlas	936	-5.93	<0.001	-38	-34	-12	26
		Inf_Temp_gyrus_post_L							50
		Parahip_Ambiens_gyrus_ant_L[a]							1
		Parahip_Subiculum_gyrus_post_L[a]							11
		Lingual_gyrus_L[a]							1
		Temp_Fusiform_cortex_post_L							244
		Temp_Occip_Fusiform_cortex_L[a]							6

		Cerebral_White_Matter_L[a]							10
		Hippocampus_L							36
		Dorsal_Cerebellum_L							326
		Ventrolateral_Cerebellum_L							214
		Pons[a]							11
		Dorsal_Cerebellum_R	101	-4.17	0.039	40	-66	-28	73
		Ventrolateral_Cerebellum_R							28
		Dorsal_Cerebellum_R	224	-4.94	<0.001	24	-70	-44	22
		Ventrolateral_Cerebellum_R							202
		outside atlas	2513	-5.90	<0.001	6	-60	-30	46
		Inf_Temp_gyrus_post_R[a]							13
		Temp_Occip_Fusiform_cortex_R							96
		Dorsal_Cerebellum_R							519
		Dorsal_Cerebellum_L							431
		Ventrolateral_Cerebellum_R							451
		Ventrolateral_Cerebellum_L							318
		Medial_Cerebellum_R							244
		Medial_Cerebellum_L							380
		Pons[a]							4
		Medulla[a]							11
		outside atlas[a]	499	-6.43	<0.001	-32	-4	-46	2
		Anterior_Temp_Pole_L							301
		Middle_Temp_gyrus_ant_L[a]							13
		Inf_Temp_gyrus_ant_L[a]							4
		Temp_Fusiform_cortex_ant_L							173
		Temp_Fusiform_cortex_post_L[a]							6

		outside atlas[a]	323	-5.84	<0.001	52	-14	-36	3
		Middle_Temp_gyrus_post_R[a]							1
		Inf_Temp_gyrus_ant_R							34
		Inf_Temp_gyrus_post_R							241
		Temp_Fusiform_cortex_post_R							44
		outside atlas[a]	201	-4.53	0.001	-52	-12	-16	2
		Insular_cortex_L							22
		Sup_Temp_gyrus_ant_L							57
		Sup_Temp_gyrus_post_L[a]							8
		Middle_Temp_gyrus_ant_L							23
		Middle_Temp_gyrus_post_L							87
		Cerebral_White_Matter_L[a]							2
		Insular_cortex_R[a]	454	-5.75	<0.001	30	2	-22	9
		Anterior_Temp_Pole_R							341
		Inf_Temp_gyrus_ant_R[a]							2
		Parahip_Ambiens_gyrus_ant_R							18
		Cerebral_White_Matter_R[a]							32
		Amygdala_R							52
		Parahip_Ambiens_gyrus_ant_L[a]	127	-5.29	0.014	-20	-6	-20	1
		Cerebral_White_Matter_L[a]							1
		Hippocampus_L							52
		Amygdala_L							73

Supplementary Table 7: All brain regions with change in activity during recovery from hypoglycaemia vs baseline in subjects with impaired awareness of hypoglycaemia (IAH)

Period compared to baseline	Response direction	Group (Region Name)	Cluster Size, *k* (voxels)	T (peak)	P (corrected cluster)	x	y	z	Subcluster Size
**RECOVERY**									
	**Increase**	**IAH**							
		Ant_Dorsolateral_Frontal_cortex_L	213	4.88	<0.001	-24	60	8	207
		Anterior_Medial_Frontal_cortex_L[a]							6
	**Decrease**	**IAH**							
		outside atlas[a]	199	-4.32	0.001	-56	-52	-22	1
		Inf_Temp_gyrus_post_L							59
		Dorsal_Cerebellum_L							51
		Ventrolateral_Cerebellum_L							88
		Inf_Temp_gyrus_post_R	148	-5.87	0.006	48	-52	-16	69
		Temp_Occip_Fusiform_cortex_R							79
		outside atlas[a]	103	-4.68	0.036	-30	-82	-28	1
		Dorsal_Cerebellum_L							77
		Ventrolateral_Cerebellum_L							25
		Dorsal_Cerebellum_R[a]	188	-5.67	0.002	28	-78	-34	15
		Ventrolateral_Cerebellum_R							173
